# Metal surface-triggered DNAzyme catalysis for efficient DNA cleavage

**DOI:** 10.1038/s42004-026-01893-z

**Published:** 2026-01-19

**Authors:** Fangning Jiang, Yan Dong, Wenqian Yu, Huiyu Tian, Longping Yang, Ziyi Jia, Yongjie Sheng, Dayong Si, Jiacui Xu, Dazhi Jiang

**Affiliations:** 1 Key Lab for Molecular Enzymology & Engineering of the Ministry of Education, School of Life Sciences, Jilin University, Changchun, China; 2 School of Life Sciences, Jilin University, Changchun, China; 3 School of Animal Sciences, Jilin University, Changchun, China

**Keywords:** Catalytic DNA, DNA

## Abstract

DNAzymes conventionally require dissolved metal ions for catalytic functions. Herein, we report that metal surfaces directly activate a self-cleaving DNAzyme (PL) at solid-liquid interfaces. PL exhibits activities on copper, vanadium and tantalum surfaces, within a minimal reaction system comprising only the metal surface, PL and double-distilled water. This interfacial activation is highly material-specific, showing complete absence of activity on plastics, glass or wood etc. Mechanistic studies reveal that dissolved oxygen could react with metal surfaces to generate superoxide anions, which serve as triggers for DNA-cleavage. The reaction shows modulatable characteristics, with inhibition by ethylenediaminetetraacetic acid, catalase, nitroblue tetrazolium and cytochrome *c*, versus enhancement by vitamin C, glutathione and catechol. Furthermore, metal surface-mediated activation was also observed in F-8, Ag10c and I-R3 DNAzymes, indicating that this phenomenon is not an isolated occurrence. This work establishes macroscopic metals as DNAzyme’s cofactors, extending DNAzyme catalysis beyond conventional homogeneous systems to heterogeneous interfacial environments.

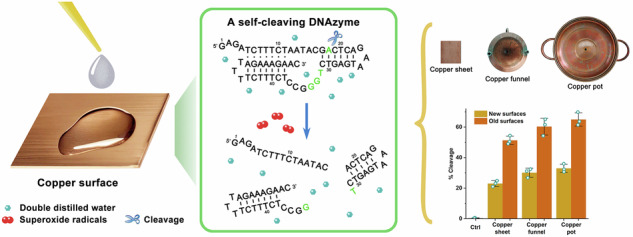

## Abstract (web summary)

DNAzymes rely on dissolved metal ions as cofactors for catalytic activity, however, the effects of metals in heterogeneous systems are not fully understood. Here, the authors demonstrate that metal surfaces can directly activate DNAzymes at solid-liquid interfaces, revealing a mechanism involving superoxide anions.

## Introduction

More than a decade after the discovery of RNAzymes (ribozymes), catalytically active single-stranded DNA molecules known as DNAzymes (deoxyribozymes, catalytic DNAs) were obtained through in vitro selection^1^. Subsequently, various types of DNAzymes have been reported, predominantly targeting RNA cleavage through metal ion coordination^2–6^. Classic examples include Mn^2+^/Mg^2+^/Ca^2+^ -dependent 10–23^7^ and 8-17^8^ DNAzymes, while specialized DNAzymes exhibit strict metal ion selectivity (e.g., Na^+^-specific NaA43^9^, Li^+^-responsive 20-4^10^,  Ag^+^-activated Ag10c^11^, Mn^2+^-dependent 11-5^12^, UO_2_^2+^-assisted 39E^13^, and trivalent lanthanides-requiring Ce13d^14^). Beyond RNA substrates, DNAzymes also cleave DNA through metal-mediated catalysis. Representative DNAzymes include Mn^2+^-dependent F-8^15^, Cu^2+^-activated RadDz3^16^, and Zn^2+^-requiring 9NL27^17^, I-R3^18^ and III-R3^19^. Functional diversity extends to phosphotransferase reactions (Zn^2+^-dependent Supernova^20^, Apollon^21^ and Aurora^22^ DNAzymes) and nitrogen-group modifications (Mg^2+^/Mn^2+^/Zn^2+^-activated N-acylation 8JB210^23^ and N-alkylation 6LA230^24^ DNAzymes). A universal feature across these DNAzymes is their obligatory requirement for dissolved metal ions as soluble cofactors.

Based on the strict metal ion dependence of DNAzymes’ activity, DNAzymes-based sensors have been developed for sensing and imaging metal ions in vivo^4,25–28^. In Zn²⁺ detection and imaging applications, Wang et al. developed a photocaged 8-17 DNAzyme activated by selenocysteine-mediated regulation, enabling spatiotemporal Zn^2+^ mapping in live cells^29^. Xiong et al. integrated DNAzymes with nano-luciferase via bioluminescent resonance energy transfer (BRET), achieving whole-body Zn^2+^ tracking in mice^30^. Liu et al. combined CRISPR/Cas9 localization with DNAzyme probes for nuclear Zn^2+^ imaging^31^. Beyond metal sensing, programmable DNAzyme recognition extends to miRNA profiling^32–34^, protein analysis^35,36^, and pathogen detection^37–39^, with therapeutic applications in gene regulation^25,40,41^. Notably, metal ions function dually in these systems - as both primary detection targets and essential cofactors^29–31^.

**Fig. 1:**
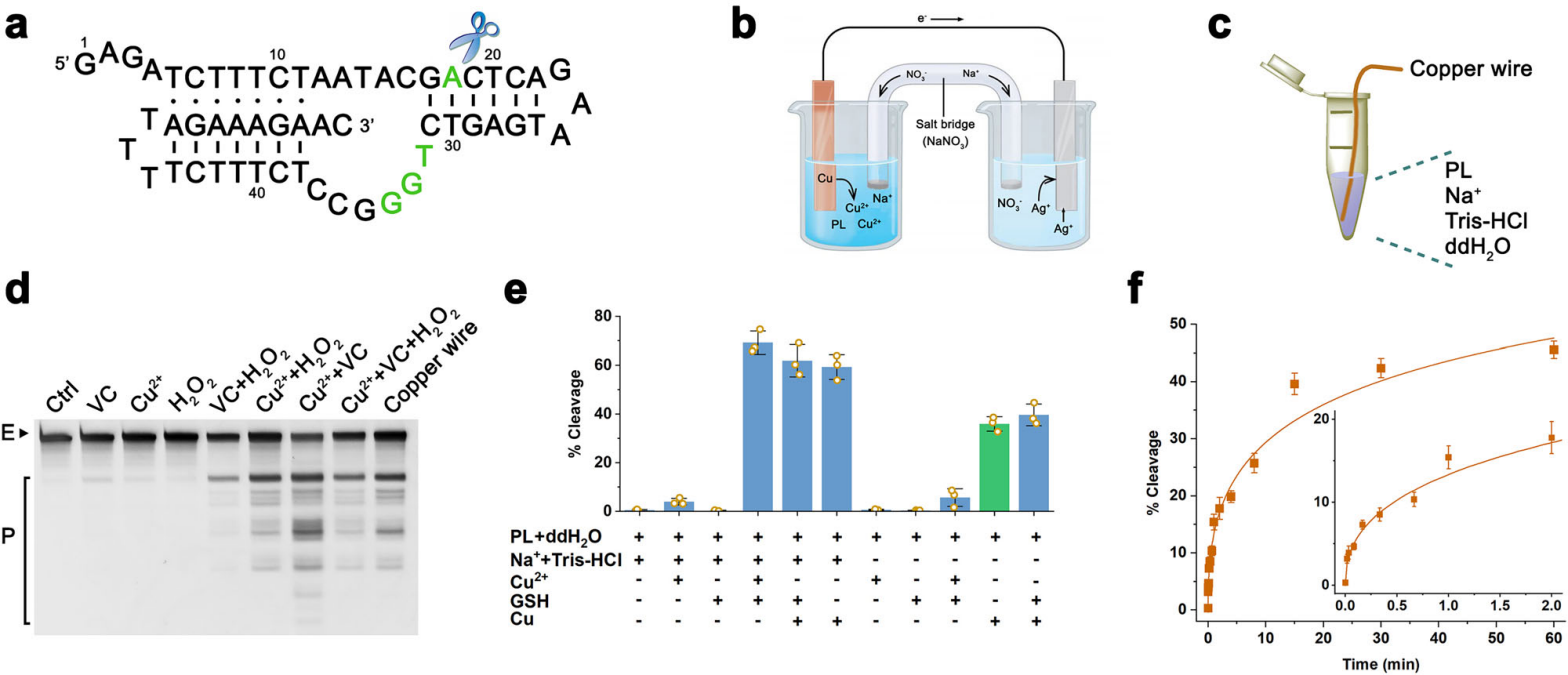
The relationship between copper wire and the PL DNAzyme. **a** The sequence and secondary structure of the PL DNAzyme. **b** Scheme of the galvanic cell**. c** Scheme of the reaction between copper wire and PL**. d** Identification of the role of copper wire. The E and P represent PL and the cleavage products, respectively. **e** Simplification of reaction system components. **f** Time course of the cleavage reaction of PL in the presence of copper wire. The error bars represented the standard deviations from three repeated measurements.

The Breaker group first identified a class II DNA-cleaving DNAzyme (PL) through structural optimization, which exhibits a pistol-like secondary structure (Fig. 1a; Supplementary Table 1)^42,43^. This metal ion-dependent DNAzyme features a characteristic three-helix junction architecture, with 3’-terminal nucleotides directly participating in catalysis^43^. While Cu^2+^ serves as its essential cofactor^44^, its activity markedly increases with redox-active molecules (enhancing factors) like vitamin C (VC) or hydrogen peroxide (H_2_O_2_)^43,45^. Our work expanded the range of enhancing factors to include thiols (reduced glutathione, 2-mercaptoethanol), phenolic (catechol, pyrogallol) and amine compounds (hydroxylamine, *o*-phenylenediamine) etc., demonstrating their capacity to mimic VC/H_2_O_2_ enhancement^46,47^. Building on this regulatory mechanism, we engineered PL-based biosensors for Cu^2+^ detection in drinking water^48^ and pickled cucumber soup^49^, and salivary glucose monitoring^50^.

Based on our previous work in allosteric DNAzyme regulation via oligonucleotide binding^51^, we devised an electrochemical strategy to modulate PL activity using a galvanic cell system (Fig. 1b). The Cu/AgNO_3_ system drives anode oxidation (Cu → Cu^2+^ + 2e^–^) and cathode reduction (Ag^+^ + e^–^ → Ag), enabling precise Cu^2+^ release for activity enhancement. Intriguingly, the control experiments revealed that copper wire (bare copper surface) alone triggered efficient DNA cleavage in aqueous solutions (Fig. 1c) - marking a phenomenon previously undocumented in DNAzyme catalysis. This discovery prompted systematic investigation of surface material effects, oxygen dependency, and reaction modulators, ultimately revealing metal interface-enabled catalysis distinct from classical solution-phase activation.

## Results

### The relationship between copper wire and the activity of PL

Based on the observation that PL-containing solutions catalyzed substrate cleavage upon contact with copper (Cu) wire, we hypothesized that metallic copper may functionally mimic PL’s cofactor. PL typically employs dual cofactor systems (e.g., Cu^2+^ + H_2_O_2_, Cu^2+^ + VC or Cu^2+^ + GSH) to enhance cleavage efficiency^43,45–47^. Experimental results revealed distinct catalytic effects among individual components: Cu^2+^ and H_2_O_2_ exhibited relatively low activity, whereas VC demonstrated slightly higher cleavage capacity. In dual cofactor systems, the Cu^2+^ + VC combination achieved the optimal performance, followed sequentially by Cu^2+^ + H_2_O_2_ and VC + H_2_O_2_ systems (Fig. 1c), consistent with previous reports^46,47^. Notably, copper wire alone displayed cleavage efficiency comparable to that of the Cu^2+^ + H_2_O_2_ dual-cofactor system and even slightly exceeded the Cu^2+^ + VC + H_2_O_2_ ternary-cofactor system, while maintaining identical cleavage site specificity (Fig. 1d; Supplementary Figs. 1, 2).

To elucidate copper’s catalytic role, we designed a simplified reaction system only containing PL and copper wire. The Cu^2+^+GSH system was taken as a control, given that GSH alone exhibits no activation effect on PL, whereas VC and hydrogen peroxide (H_2_O_2_) show minimal activities, respectively^47,48^. Consistent with previous reports, the optimal cleavage activity required neutral pH and sodium ions (Na^+^), likely through pH-dependent activation of PL and Na^+^-mediated structural stabilization of PL’s catalytic domain. Notably, although buffer components and Na⁺ significantly increased PL’s activity in standard reaction systems, the copper wire-based catalytic system exhibited a detectable DNA cleavage activity even in ddH₂O, in the absence of pH buffers or Na^+^ (Fig. 1e; Supplementary Fig. 3). To reduce confounding variables in mechanistic investigations, we established a minimal reaction system comprising only PL, metallic copper, and ddH₂O. Kinetic analysis revealed rapid copper-dependent activation, with cleavage products detectable within 1 s of copper exposure and exhibiting time-dependent progression over 15 min (Fig. 1f; Supplementary Fig. 4). Beyond this timepoint, reaction kinetics essentially stabilized, identifying 15 min as the optimal incubation time.

### The effects of metal and non-metal surfaces on the activity of PL

**Fig. 2:**
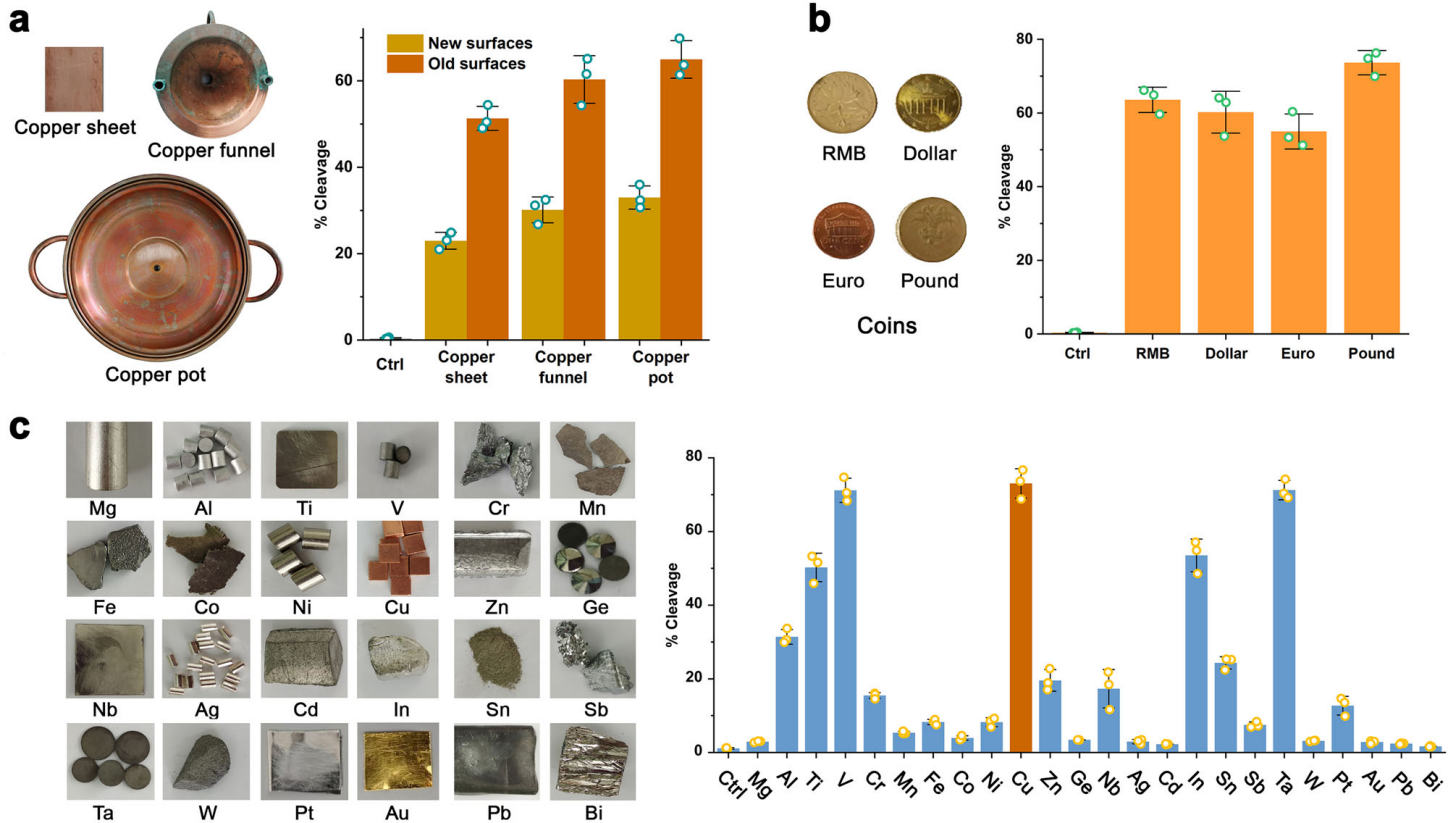
The effects of metal and non-metal surfaces on PL catalysis. **a** The effects of copperwares on PL catalysis. **b** The effects of copper coin surfaces on PL catalysis. **c** The effects of 24 types of metal surfaces on PL catalysis. Composition of the reaction system: 0.1 μM PL, metal samples, and ddH_2_O. The reaction temperature and time were set at 23 °C and 15 min, respectively. The error bars represented the standard deviations from three repeated measurements.

We further investigated the catalytic effects of various copper-based materials (sheets, funnels, and pots) on PL activity. Initial hypotheses suggested that surface oxide layers formed during ambient exposure might inhibit PL-mediated cleavage. To test this, we compared aged interfaces with freshly polished surfaces (generated by sandpaper abrasion). Contrary to expectations, all three copper forms exhibited comparable cleavage efficiencies, with newly polished interfaces demonstrating 18–23**%** lower activity than oxidized surfaces (Fig. 2a; Supplementary Fig. 5). Given copper’s widespread use in coinage due to its corrosion resistance, we examined the influence of copper composition in four circulation coins (RMB, US Dollar, Euro and British Pound) on PL’s activity. All coins effectively facilitated PL-catalyzed cleavage. The catalytic efficiency ranking was: Pound > RMB > Dollar > Euro (Fig. 2b; Supplementary Fig. 6).

Surface-area dependency analysis revealed a positive correlation between copper-PL interfacial contact and its catalytic cleavage efficiency. Quantitative characterization of 100 μL PL droplets on copper coins showed contact diameters of 8.0 ± 0.2 mm (area: 50.5 ± 3.2 mm²; Supplementary Table 2). Theoretical calculations suggested that achieving equivalent contact areas would require only 747 ng of 10-nm or 7.47 μg of 100-nm copper nanoparticles. Experimentally, we modulated interfacial contact by varying the number of copper wires (commercially sourced electrical conductors) in the reaction system. Nonlinear regression analysis using the Hill model showed wire-number-dependent cleavage rate (1–32 wires), with saturation above the threshold of 8 wires (Supplementary Fig. 7).

To evaluate the material specificity, we analyzed 24 types of metals for PL activation (Supplementary Table 3). Three-tiered catalytic performance emerged. High activity: Cu, Ta, V (≥70% cleavage), Moderate activity: In, Ti, Al, Sn, Zn, Nb, Cr, Pt (20–55% cleavage), Low or negligible activity: Fe, Ni, Mn, Sb, Co, Ge, W, Ag, Mg, Au, Pb, Cd, Bi (<10% cleavage) (Fig. 2c). Crucially, all active metals produced identical cleavage patterns (Supplementary Fig. 8), indicating conserved catalytic mechanisms. We further compared metals with 10 types of non-metallic materials (Supplementary Table 4): cellulose-based (paper, wood), silica-based (glass, quartz), ceramic (ceramic cup lip), calcium carbonate (marble) and polymers (plastic wrap, nitrile gloves, foam boards, paint). Experimental data conclusively demonstrated that none of the ten non-metallic materials exhibited detectable self-cleavage activity (Supplementary Fig. 9).

### Inhibitory factors of PL-catalyzed reactions

**Fig. 3:**
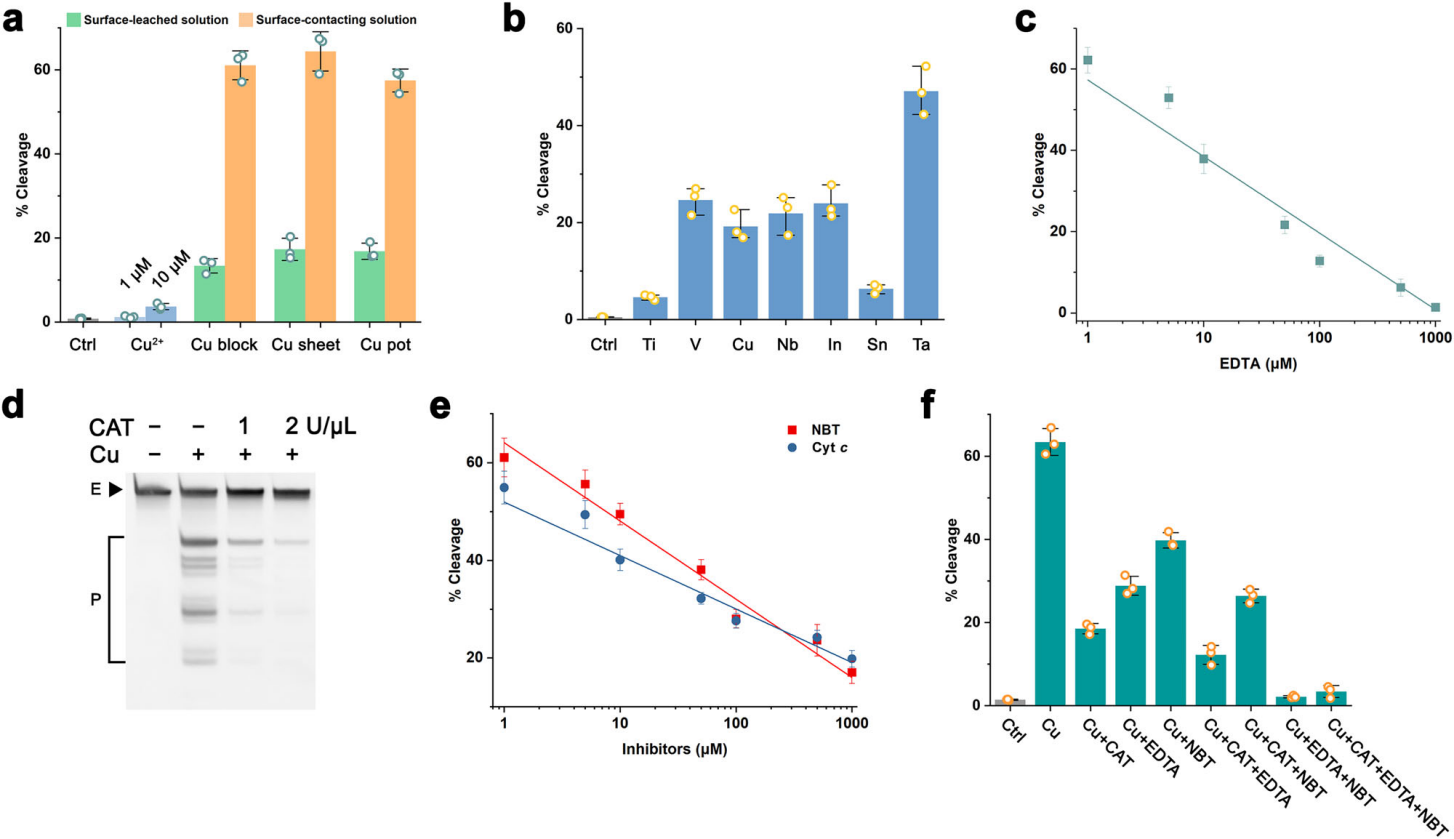
Identification and analysis of PL inhibitors. **a** Comparative impacts of copper surface-leached and surface-contacting solutions. **b** Comparative effects of seven metal surface-leached solutions. **c** Chelation effect of EDTA on surface-contacting solutions. **d** Hydrogen peroxide scavenging by CAT in Cu surface-contacting solutions. The E and P represent PL and the cleavage products, respectively. **e** Inhibitory effects of NBT and Cyt *c*. **f** Combined effects of inhibitory factors. The error bars represented the standard deviations from three repeated measurements.

To investigate metal surface activation mechanisms, we hypothesized that trace metal ions leaching during the material contact might mediate PL catalysis. ICP-MS analysis of aqueous solutions exposed to Cu, Ta, V, and In surfaces revealed ion release: Cu: 5.592 μM (355.641 ppb), Ta: 3.716 μM (1020.735 ppb), In: 3.652 μM (419.201 ppb) and V: 3.066 μM (156.369 ppb) (Supplementary Table 5). Comparative testing of three copper forms (block, sheet, pot) revealed that surface-leached solutions (recovered ddH_2_O from metal surfaces after immersion) exhibited 3.6- to 4.7-fold higher activity than 10 μM Cu^2+^ solutions, and direct surface-contacting solutions showed 3.4- to 4.6-fold greater efficiency than surface-leached solutions (Fig. 3a; Supplementary Fig. 10). Extended analysis of seven metal leachates (Ti, V, Nb, In, Sn, Ta, Cu) under 15 min incubation revealed catalytic capacity following the hierarchy: Ta > V > In > Nb > Cu > Sn > Ti (Fig. 3b; Supplementary Fig. 11). In contrast, Cu^2+^ concentrations ranging from 1 to 10 μM demonstrated marginal cleavage activities (<5%), while Fe^2+^, Zn^2+^, and In^3+^ exhibited no detectable cleavage activity over the concentration range of 1–1000 μM (Supplementary Fig. 12). To investigate the role of metal ions released from metal surfaces, we introduced ethylenediaminetetraacetic acid (EDTA) to chelate Cu^2+^ and suppress PL activity. Results showed a progressive decline in PL catalytic activity with increasing EDTA concentrations (Fig. 3c and Supplementary Fig. 13). These findings suggest that metal ions can be leached from metal surfaces into the aqueous phase, which alone are insufficient to drive efficient PL cleavage, implying the existence of a critical missing component in the reaction mechanism.

To elucidate the catalytic mechanism, we systematically probed reactive oxygen species (ROS) involvement in PL activation. PL commonly uses the dual cofactor systems Cu^2+^ + VC or Cu^2+^ + H_2_O_2_, which can undergo Fenton-like reactions and produce hydroxyl radicals (HO•). Since 3,3’,5,5’-tetramethylbenzidine (TMB) is often used to eliminate HO•, we used TMB as a scavenger for HO• to detect the impact of HO• on the activity of PL. TMB scavenging assays detected no activity reduction (Supplementary Fig. 14). Catalase (CAT) supplementation (1 and 2 U per μL) in copper surface-contacting solutions decreased cleavage by 49% and 57% (Fig. 3d; Supplementary Fig. 15), revealing endogenous H_2_O_2_ generation. Exogenous H_2_O_2_ alone resulted in less than 8% cleavage efficiency (Supplementary Fig. 16), in contrast to the 57–64% efficiency observed in copper surface-contacting solutions (Fig. 3a), indicating H₂O₂ acts as a secondary modulator rather than a primary effector.

We subsequently systematically evaluated the role of superoxide anion (O_2_^•–^) in the catalytic system by using nitroblue tetrazolium (NBT) and cytochrome *c* (Cyt *c*) as O_2_^•–^ scavengers. Their inhibitory effects on the PL-catalyzed reactions were quantitatively assessed. Concentration-response analysis revealed a progressive attenuation of PL catalytic activity (61 → 17% cleavage) corresponding to NBT-mediated superoxide (O₂•⁻) scavenging efficiency (Fig. 3e, Supplementary Fig. 17), thereby mechanistically implicating O₂•⁻ involving in the catalytic process. Parallel inhibition studies with Cyt *c* revealed similar dose-dependent inhibition profiles (Fig. 3e, Supplementary Fig. 18), thereby confirming the essential role of O_2_^•–^ in PL-mediated cleavage.

Following characterization of individual inhibition profiles we pursued combinatorial inhibition approaches. Notably, the NBT + EDTA dual treatment demonstrated marked attenuation of PL activity (63 → 2% cleavage) (Fig. 3f, Supplementary Fig. 19). To validate the universality of these inhibition mechanisms, we systematically evaluated vanadium (V) and tantalum (Ta) metal systems. Experimental data confirmed that the identified inhibitor combinations effectively attenuated catalytic activity across these metal variants (Supplementary Figs. 20, 21), demonstrating the generalizability of superoxide-mediated catalytic pathways in metal surface-dependent PL catalysis.

### The effect of dissolved oxygen on PL catalysis

**Fig. 4:**
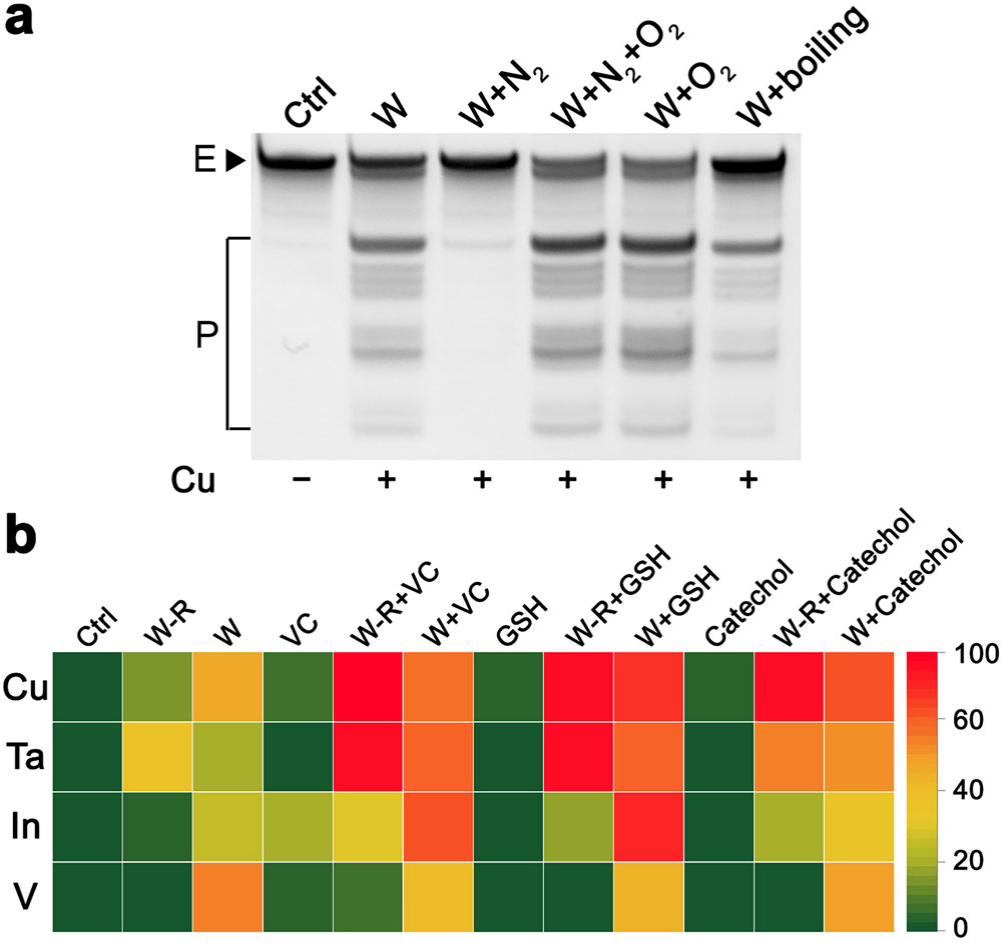
Dissolved oxygen effect and PL’s enhancing factors. **a** The effect of dissolved oxygen in water. The E and P represent PL and the cleavage products, respectively. **b** Identification of PL’s enhancing factors. W-R indicates metal (Cu, Ta, In, V) surface-leached solutions, and W represents metal (Cu, Ta, In, V) surface-contacting solutions.

Our experimental evidence indicates that O_2_^•–^ likely serves as a critical intermediate in the PL cleavage mechanism. To address its origin, we considered dissolved oxygen in aqueous solutions – a frequently underestimated background factor in solution-phase reactions. We hypothesize that dissolved oxygen undergoes surface-mediated redox reactions with the metallic component to generate O_2_^•–^. This proposed mechanism suggests that PL activity could be modulated by oxygen depletion to suppress O_2_^•–^ formation. To test this hypothesis, we employed thermal degassing (boiling) and inert gas purging (N_2_) to create oxygen-depleted aqueous environments for PL catalysis. As demonstrated in Fig. 4a and Supplementary Fig. 22, the boiling pretreatment reduced but did not eliminate the catalytic activity, with residual product formation indicating persistent dissolved oxygen content after treatment. In contrast, nitrogen-purged systems exhibited near-complete suppression of PL activity. Crucially, reintroduction of oxygen to nitrogen-purged systems restored catalytic performance to Cu surface levels (W) – even exceeding baseline activity in some cases. These findings identified dissolved oxygen as an essential reactant in PL catalysis, where its reduction to O_2_^•–^ constitutes a critical step in the catalytic cycle.

### Enhancing factors of PL-catalyzed reactions

Following the identification of PL’s catalytic inhibitors, we explored potential enhancing factors. Based on previous studies of PL’s cofactors^46,47^, we investigated compounds known to enhance copper-mediated catalysis. While Cu²⁺ alone exhibited limited PL cleavage activity, significant rate enhancement was observed upon addition of vitamin C (VC), reduced glutathione (GSH), cysteine, 2-mercaptoethanol, dithiothreitol, catechol, resorcinol, hydroquinone, pyrogallol, hydroxylamine or *o*-phenylenediamine etc. We evaluated three primary enhancing factors (VC, GSH, catechol) across four metallic cofactor systems (Cu, Ta, In, V). In Cu-contacting solutions, all three compounds enhanced PL activity, with GSH demonstrating moderately stronger effects than VC and catechol (Fig. 4b, Supplementary Fig. 23). This enhancement became more pronounced in Cu surface-leached solutions, following the efficacy order: GSH > VC > catechol (Fig. 4b). The relationship between copper leaching time and PL DNAzyme activity is shown in Supplementary Fig. 24. The cleavage activity of the PL DNAzyme increased with leaching time, although the overall cleavage extent of PL DNAzyme remained relatively low, reaching a maximum of only ~11%. However, the addition of GSH to the leached solution significantly enhanced the PL DNAzyme activity. At a leaching time of 60 min, the cleavage extent of PL DNAzyme reached 64%. This behavior is similar to the relationship between PL DNAzyme activity and Cu^2+^ or Cu^2+^ + GSH^46,47^.

Ta-based systems exhibited distinct activation patterns: VC showed the strongest enhancement in both Ta surface-contacting and Ta surface-leached solutions, followed by GSH and catechol. For indium (In) cofactors, VC and GSH enhanced activity in In surface-contacting solutions, while catechol showed no effect. Intriguingly, all three enhancing factors improved performance in In surface-leached solutions, ranked as VC > GSH > catechol. Vanadium systems displayed unique behavior – no enhancement occurred in V surface-contacting solutions, while only VC enhanced PL activity in V surface-leached solutions. Our previous studies identifying the critical role of GSH’s thiol group^47,48^ suggest that other thiol-containing compounds (e.g., cysteine, dithiothreitol, 2-mercaptoethanol) can similarly enhance PL activity. Structural analogs of catechol, including dopa, dopamine, chlorogenic acid and caffeic acid, are predicted to show similar enhancement effects.

## Discussion

The efficient activation of PL catalysis by copper surfaces is mediated through a redox cycling mechanism involving copper species. Upon contact with the copper surface, trace amounts of Cu^2+^ are released into the aqueous solution via oxidation (Eq. (1)). These Cu^2+^ ions subsequently undergo redox equilibrium with metallic copper to generate Cu^+^ ions (Eq. (2)), representing the reverse of Cu^+^ disproportionation. A critical catalytic step occurs when Cu^+^ reacts with dissolved oxygen (O_2_) to regenerate Cu^2+^ while producing superoxide radicals (O_2_^•–^) (Eq. (3))^52^. These radicals subsequently disproportionate into hydrogen peroxide (H_2_O_2_) and O_2_ (Eq. (4)), while H_2_O_2_ further reacts with Cu^2+^ to reform Cu^+^ and additional O_2_^•–^ (Eq. (5))^46,47^. This cyclic process (Eqs. (3)–(5)) sustains the catalytic cleavage activity observed in copper-containing systems and explains why catalase (CAT) inhibits the reaction through H_2_O_2_ decomposition.

The mechanism is modulated by multiple factors: EDTA suppresses activity by chelating Cu^2+^ ions, thereby interrupting the catalytic cycle, while NBT and Cyt *c* act as O_2_^•–^ scavengers that neutralize the essential radical species. Although dissolved oxygen is frequently overlooked in enzymatic systems, its removal effectively halts the reaction cascade by preventing the oxygen-dependent step in Eq. (3). Conversely, the GSH enhances catalysis by reducing Cu^2+^ to Cu^+^ (Eq. (6))^53–56^, amplifying the Cu^+^/O_2_ redox cycle and increasing O_2_^•–^ production. The central role of O_2_^•–^ in this catalytic system enables potential expansion beyond metallic surfaces. Photocatalytic materials like covalent organic frameworks (COFs)^57^, which generate O_2_^•–^ under illumination, could substitute metal surfaces as PL cofactors.

1 $${{{\rm{Cu}}}}\to {{{{\rm{Cu}}}}}^{2+}+{2}^{{{{\rm{e}}}}{{{\rm{\hbox{-}}}}}}$$

2 $${{{\rm{Cu}}}}+{{{{\rm{Cu}}}}}^{2+}\leftrightarrow {{{{\rm{Cu}}}}}^{+}$$

3 $${{{{\rm{Cu}}}}}^{+}+{{{{\rm{O}}}}}_{2}\to {{{{\rm{Cu}}}}}^{2+}+{{{{{\rm{O}}}}}_{2}}^{{{\bullet }}-}$$

4 $${{2{{{\rm{O}}}}}_{2}}^{{{\bullet }}{-}}+{2{{{\rm{H}}}}}^{+}\to {{{{\rm{H}}}}}_{2}{{{{\rm{O}}}}}_{2}+{{{{\rm{O}}}}}_{2}$$

5 $${{{{\rm{Cu}}}}}^{2+}+{{{{\rm{H}}}}}_{2}{{{{\rm{O}}}}}_{2}\to {{{{\rm{Cu}}}}}^{+}+{2{{{\rm{H}}}}}^{+}+{{{{{\rm{O}}}}}_{2}}^{{{\bullet }}-}$$

6 $${{{{\rm{Cu}}}}}^{2+}+2{{{\rm{GSH}}}}\to {{{{\rm{Cu}}}}}^{+}+{{{\rm{GS}}}}-{{{\rm{SG}}}}+{2{{{\rm{H}}}}}^{+}$$

The system’s inherent superoxide anion dependence suggests two distinct biomedical applications: (1) Engineering PL-based probes for intracellular O_2_^•–^ monitoring^58,59^, leveraging its native radical responsiveness, and (2) Developing biocompatible O_2_^•–^ scavengers for therapeutic intervention in oxidative stress-related pathologies. These applications capitalize on PL’s dual functionality as both a sensor and modulator of reactive oxygen species, combined with its inherent biological compatibility. Additionally, site-specific chemical modification of PL and its substrate could facilitate the development of fluorescent probes for imaging the spatial distribution of copper nanomaterials within cells.

In addition to the close correlation between PL DNAzyme activity and free radicals, the catalytic activities of other oxidative DNAzymes, such as F-8^15^ and RadDz3^60^, are also influenced by free radicals. This is evidenced by the significant enhancement of their cleavage activities upon H_2_O_2_ addition, a response consistent with that of the PL DNAzyme^46,47^. The cleavage of the RadDz3 DNAzyme is induced by superoxide anion attacking the C4 position of the deoxyribose in guanosine deoxyribonucleotide^60^. Given the observed correlation between PL DNAzyme activity and aqueous solutions derived from metal surfaces, we speculate that other DNAzymes with oxidative DNA cleavage activity may also exhibit ‘metal surface-activated activity’. This hypothesis is supported by the findings that the F-8 DNAzyme similarly demonstrated manganese (Mn) surface-dependent catalytic behavior, and the addition of VC enhanced its cleavage efficiency both in aqueous solution and reaction buffer (50 mM HEPES, pH 7.4). However, the Mn^2+^+VC system enhanced activity only in the reaction buffer, not in aqueous solution (Supplementary Fig. 25).

Having confirmed that metal surfaces activate the oxidative cleavage activity of DNAzymes, we further investigated whether metals could also trigger the hydrolytic cleavage activity toward RNA and DNA—a more common function among DNAzymes. We selected the Ag10c DNAzyme^11^, known for its high specificity toward Ag^+^ and RNA-hydrolyzing activity, and the I-R3 DNAzyme^18^, which is strictly Zn^2+^-dependent and possesses DNA-hydrolyzing capability. Experimental data demonstrated that metallic silver (Ag) surfaces activated the catalytic activity of the Ag10c DNAzyme (Supplementary Fig. 26), while metallic zinc (Zn) similarly triggered I-R3 activity (Supplementary Fig. 27). Since both Ag10c and I-R3 DNAzymes possess metal ion-dependent hydrolytic activity, the metal ions leached from the metal surfaces may be the most critical activating factor. As shown in Supplementary Fig. 27, the cleavage extent of the I-R3 DNAzyme on a Zn surface (46%) was even higher than that in the presence of 50 mM Zn^2+^ (30%). It is unlikely that the concentration of metal ions leaching from the Zn surface into the aqueous solution would reach significant levels within 60 min at room temperature. However, near the Zn metal–liquid interface, leached zinc ions may accumulate, forming a locally high concentration that efficiently activates the I-R3 DNAzyme.

The consistent activation of PL, F-8, Ag10c, and I-R3 DNAzymes by metal surfaces implies a potential generality of this phenomenon across DNAzymes. We further hypothesize that such ‘metal surface-activated activity’ may extend to other catalytic nucleic acids, including RNAzyme (ribozyme)^61^, XNAzyme^62^, FANAzyme^63^ and TNAzyme^64^ etc. While these DNAzymes share key operational features with nanozymes^65^ at the solid-liquid interface, their catalytic architectures diverge fundamentally: nanozymes rely on solid-phase nanoparticles to mimic enzymatic activities, whereas interface-reactive DNAzymes harness macroscale metal surfaces as catalytic cofactors. This discovery opens opportunities for in vitro screening approaches targeting DNAzymes with enhanced metal surface compatibility, potentially yielding catalysts optimized for solid-liquid interface reactions.

## Methods

### Materials

The DNAzymes were synthesized by Sangon Biotech Co., Ltd. (Shanghai, China) and underwent purification through ULTRAPAGE or HPLC. GelRed was purchased from Biotium Inc. The metals and a variety of other reagents used were of analytical reagent grade. Double distilled water (ddH_2_O) was used throughout the experiments.

### PL cleavage assay

The cleavage assay was performed in 100 μL reaction solution containing 0.1 μM PL and ddH_2_O on the metal surfaces. The reaction mixture was incubated at room temperature (~23 °C) for 15 min and then terminated by adding precipitants (200 μL of 100% ethanol, 20 μL of 3 M NaOAc (pH 5.2), 1 μL of 10 mg per mL glycogen) for precipitation at −20 °C for 10 min. The pellet was collected by centrifugation (Hermle Z323K) for 10 min at 14,000 rpm. The pellet obtained was further washed with 70% ethanol and subjected to centrifugation for 5 min at 14000 rpm. Supernatant was discarded and the pellet was air dried. The dried pellet was dissolved in 10 μL double distilled water, and an equal volume of the 2× loading buffer (8 M urea, 20 mM EDTA, 50 mM Tris-HCl (pH 7.5), 0.25‰ xylene cyanol FF, and 0.25‰ bromophenol blue) is added. The DNA solution was separated by 16% denaturing polyacrylamide gel electrophoresis. Gel was stained with 3× GelRed solution for 10 min and visualized by UV *trans*-illumination (Clinx GenoSens 1880).

### Reporting summary

Further information on research design is available in the Nature Portfolio Reporting Summary linked to this article.

## Supplementary information

Supplementary Information

Description of Additional Supplementary Files

Supplementary Data 1

Reporting summary

## Data Availability

All data supporting the findings of this study are available within the paper and Supplementary Information. Source data used to generate figures is provided in Supplementary Data 1.
