# Supplementary material for: Metal surface-triggered DNAzyme catalysis for efficient DNA cleavage
Source: Commun Chem. 2026 Jan 19;9:91. doi: 10.1038/s42004-026-01893-z (PMC12909895; doi:10.1038/s42004-026-01893-z)
Supplement: Supplementary file 1 — Supplementary Information [file 42004_2026_1893_MOESM1_ESM.pdf]

Supporting Information

Metal surface-triggered DNAzyme catalysis for efficient DNA cleavage

Fangning Jiang[1,4], Yan Dong[1,4], Wenqian Yu[1,4], Huiyu Tian[1], Longping Yang[1], Ziyi Jia[1], Yongjie Sheng[1], Dayong Si[2], Jiacui Xu[3,*], Dazhi Jiang[1,*]

[1]Key Lab for Molecular Enzymology & Engineering of the Ministry of Education, School of Life Sciences, Jilin University, 2699# Qianjin Street, Changchun 130012, China. [2]School of Life Sciences, Jilin University, 2699# Qianjin Street, Changchun 130012, China. [3]School of Animal Sciences, Jilin University, 5333# Xi'an Road, Changchun 130062, China. [4]These authors contributed equally: Fangning Jiang, Yan Dong, Wenqian Yu. *e-mail address: jcxu@jlu.edu.cn; jiangdz@jlu.edu.cn

Keywords:

Cleavage; Copper; DNAzyme; Metal surface

Table of Contents

Supplementary Table 1. Oligonucleotide sequences of DNAzymes....................2

Supplementary Table 2. The contact area between the droplet and the copper sheet....................2

Supplementary Table 3. Metal samples and their purities....................2

Supplementary Table 4. Non-metal samples and main components....................3

Supplementary Table 5. Metal concentrations in water....................3

Supplementary Figure 1. The effect of copper wires on the PL DNAzyme....................3

Supplementary Figure 2. The full, uncropped gel image (the left part) of Figure 1d....................4

Supplementary Figure 3. The effect of reaction system components on the PL DNAzyme....................4

Supplementary Figure 4. The effect of reaction time on the PL DNAzyme....................4

Supplementary Figure 5. The effect of copper products on the PL DNAzyme....................5

Supplementary Figure 6. The effect of copper coins on the PL DNAzyme....................5

Supplementary Figure 7. The correlation between the number of copper wires and the activity of PL......5

Supplementary Figure 8. The effect of 24 different metal surfaces on the PL DNAzyme....................6

Supplementary Figure 9. The effect of 10 different none-metal surfaces on the PL DNAzyme....................6

Supplementary Figure 10. The effect of surface-leached (-contacting) solutions on copper surfaces....7

Supplementary Figure 11. Comparative effects of seven metal surface-leached solutions....................7

Supplementary Figure 12. The effect of Cu^{2+}, Fe^{2+}, In^{3+} and Zn^{2+} on the PL DNAzyme....................8

Supplementary Figure 13. The inhibition effect of EDTA on the PL DNAzyme....................8

Supplementary Figure 14. The effect of TMB on the PL DNAzyme....................8

Supplementary Figure 15. The full, uncropped gel image (the left part) of Figure 3d....................9

Supplementary Figure 16. The effect of H_2O_2 on the PL DNAzyme....................9

Supplementary Figure 17. The inhibition effect of NBT on the PL DNAzyme....................9

Supplementary Figure 18. The inhibition effect of Cyt *c* on the PL DNAzyme....................10

Supplementary Figure 19. The effect of Cu inhibitors on the PL DNAzyme....................10

Supplementary Figure 20. The effect of V inhibitors on the PL DNAzyme....................10

Supplementary Figure 21. The effect of Ta inhibitors on the PL DNAzyme....................11

Supplementary Figure 22. The full, uncropped gel image (the left part) of Figure 4a....................11

Supplementary Figure 23. The effect of enhancers on the PL DNAzyme....................12

Supplementary Figure 24. Time course of the cleavage reaction of PL in Cu surface-leached solution...12

Supplementary Figure 25. The effect of Mn surface on the F-8 DNAzyme....................13

Supplementary Figure 26. The effect of Ag surface on the Ag10c DNAzyme....................13

Supplementary Figure 27. The effect of Zn surface on the I-R3 DNAzyme....................14

Supplementary Table 1. Oligonucleotide sequences of DNAzymes

ID.	Sequences (5'→3')
PL	GAGATCTTTCTAATACGACTCAGAATGAGTCTGGGCCTCTTTCTTTTAGAAAGAAC
F-8	TGGCGTGGAAGAGGCAACCTCAGGGTCCGTATTTTATACTGCGCCAGGGTTTTCCC
Ag10c	GCCATCTTTAGGTGATTTCCACGATTATGCGGAAACAGGGCAGCGTATAGTTTTACTA TrAGGAAGATGGCGAAGC
I-R3	GACGATCTAGTTGAGCTGTCTGCATTTTGCAGACGTTGAAGGATCGTC

The red letter "rA" denotes adenine ribonucleic acid.

Supplementary Table 2. The contact area between the droplet and the copper sheet

	1	2	3	4	5	6	7	8	Mean	SD
Diameter (mm)	8.1	8.0	8.0	7.5	8.2	7.7	8.0	8.1	8.0	0.2
Area (mm^2)	51.6	52.3	52.3	44.2	52.8	46.6	52.3	51.6	50.5	3.2

Supplementary Table 3. Metal samples and their purities

Metal	Symbol	Atomic weight	Purity (%)
Magnesium	Mg	24.3	99.999
Aluminium	Al	27.0	99.999
Titanium	Ti	47.9	99.995
Vanadium	V	51.0	99.95
Chromium	Cr	52.0	99.9
Manganese	Mn	55.0	99.7
Iron	Fe	55.9	99.9
Cobalt	Co	59.0	99.8
Nickel	Ni	58.7	99.9
Copper	Cu	63.6	99.9999
Zinc	Zn	65.4	99.999
Germanium	Ge	72.6	99.99
Niobium	Nb	93.0	99.9
Silver	Ag	107.9	99.9
Cadmium	Cd	112.4	99.999
Indium	In	114.8	99.99
Stannum	Sn	118.7	99.998
Stibium	Sb	121.8	99.999
Tantalum	Ta	181.0	99.9
Tungsten	W	183.8	99.9
Platinum	Pt	195.1	99.99
Gold	Au	197.0	99.99
Lead	Pb	207.2	99.99
Bismuth	Bi	209.0	99.99

Supplementary Table 4. Non-metal samples and main components

Name	Main components
Paper	Plant fiber
Wooden stick	Lignin
Glass slide	$Na_2O \cdot CaO \cdot 6SiO_2$
Quartz cuvette	SiO_2
Ceramic cup lid	$Al_2O_3 \cdot 2SiO_2 \cdot H_2O$, SiO_2, $K_2O \cdot Al_2O_3 \cdot 6SiO_2$
Marble	$CaCO_3$
Plastic wrap	Polyethylene
Nitrile gloves	Nitrile butadiene rubber
Foam board	Polystyrene
Paint	Synthetic resin

Supplementary Table 5. Metal concentrations in water

Metal	Concentration (ppb)	Concentration (μM)
Cu	355.641	5.592
Ta	1020.735	3.716
In	419.201	3.652
V	156.369	3.066

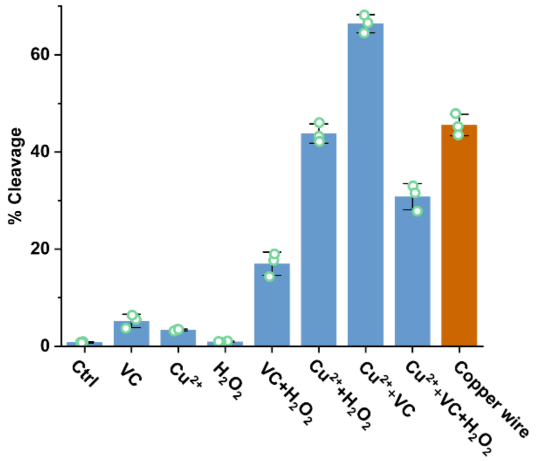

Supplementary Figure 1. The effect of copper wire on the PL DNAzyme. In the 100 μL PL-catalyzed reaction system, the concentrations of PL, VC, Cu^{2+}, H_2O_2, NaCl and Tris-HCl (pH 7.0) were 0.1 μM, 100 μM, 100 μM, 100 μM, 100 μM, 300 mM and 50 mM, respectively. The reaction temperature and time were set at 23 °C and 15 min, respectively. The error bars represented the standard deviations from three repeated measurements.

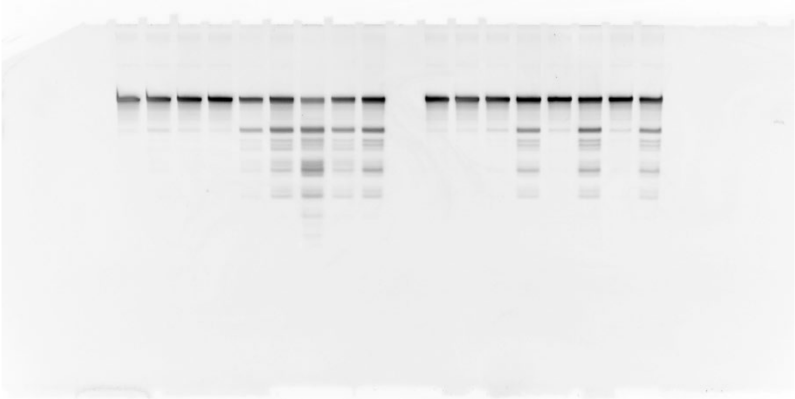

Supplementary Figure 2. The full, uncropped gel image (the left part) of Figure 1d.

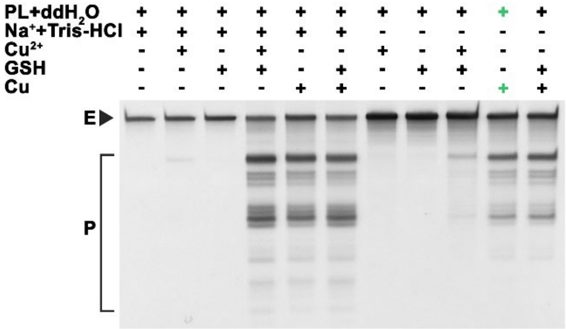

Supplementary Figure 3. The effect of reaction system components on the PL DNAzyme. In the 100 μL PL-catalyzed reaction system, the concentrations of PL, GSH, Cu^{2+}, NaCl and Tris-HCl (pH 7.0) were 0.1 μM, 100 μM, 100 μM, 300 mM and 50 mM, respectively. The reaction temperature and time were set at 23 °C and 15 min, respectively. The E and P represent PL and the cleavage products, respectively.

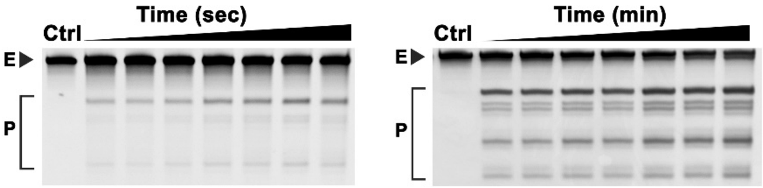

Supplementary Figure 4. The effect of reaction time on the PL DNAzyme. Composition of the 100 μL reaction system: 0.1 μM PL, Cu wire and ddH_2O. The reaction temperature was 23 °C, and the reaction times (left panel) were 1, 2, 5, 10, 20, 40 and 60 sec, while the reaction times (right panel) were 1, 2, 4, 8, 15, 30 and 60 min. The E and P represent PL and the cleavage products, respectively.

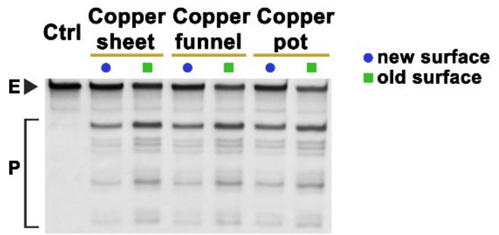

Supplementary Figure 5. The effect of copper products on the PL DNAzyme. Composition of the 100 μL reaction system: 0.1 μM PL, Cu sheet (or Cu funnel, Cu pot) and ddH_2O. The reaction temperature and time were set at 23 °C and 15 min, respectively. The E and P represent PL and the cleavage products, respectively.

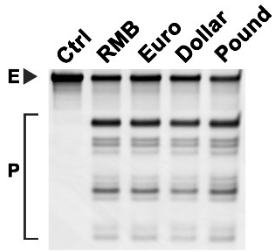

Supplementary Figure 6. The effect of copper coins on the PL DNAzyme. Composition of the 100 μL reaction system: 0.1 μM PL, coins (RMB, Euro, Dollar and Pound) and ddH_2O. The reaction temperature and time were set at 23 °C and 15 min, respectively. The E and P represent PL and the cleavage products, respectively.

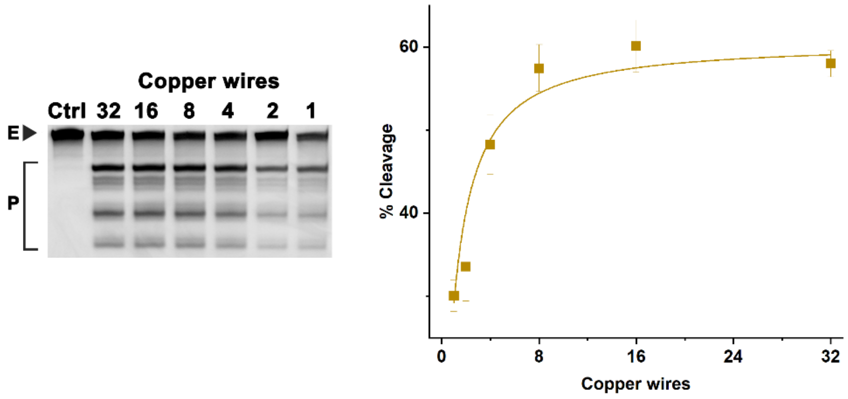

Supplementary Figure 7. The correlation between the number of copper wires and the activity of PL. Composition of the100 μL reaction system: 0.1 μM PL, Cu wires and ddH_2O. The reaction temperature and time were set at 23 °C and 15 min, respectively. The E and P represent PL and the cleavage products, respectively. The error bars represented the standard deviations from three repeated measurements.

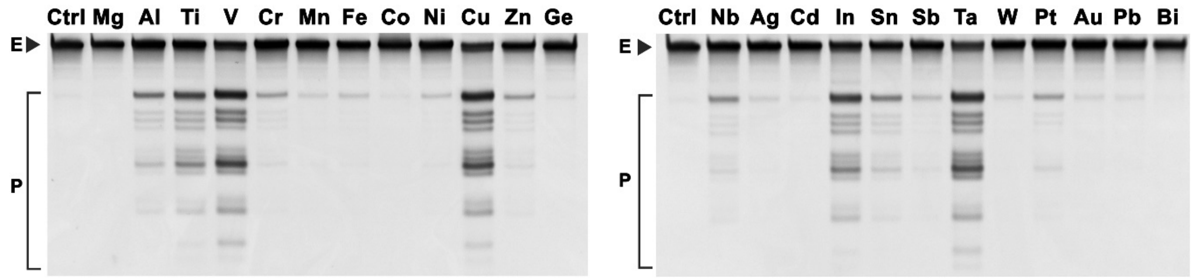

Supplementary Figure 8. The effect of 24 different metal surfaces on the PL DNAzyme. Composition of the 100 μL reaction system: 0.1 μM PL, metal surface and ddH_2O. The reaction temperature and time were set at 23 °C and 15 min, respectively. The E and P represent PL and the cleavage products, respectively.

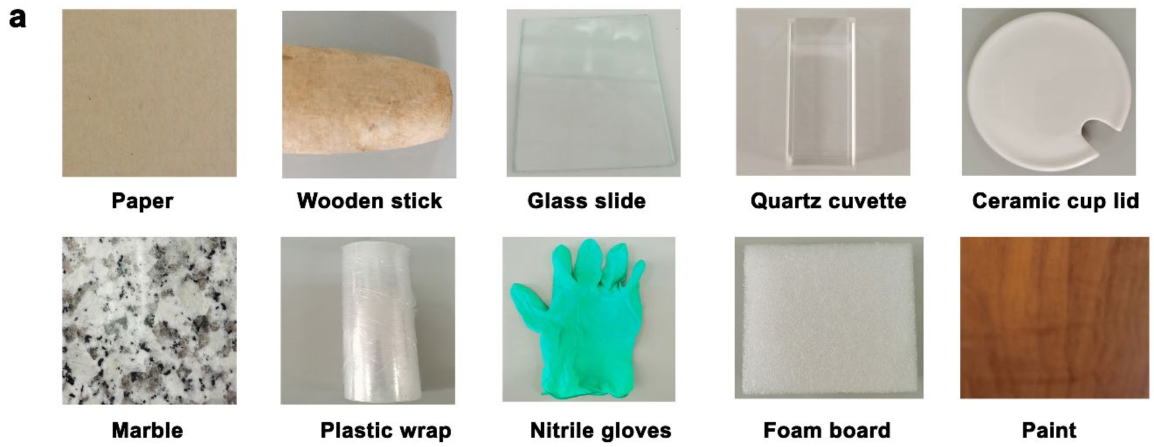

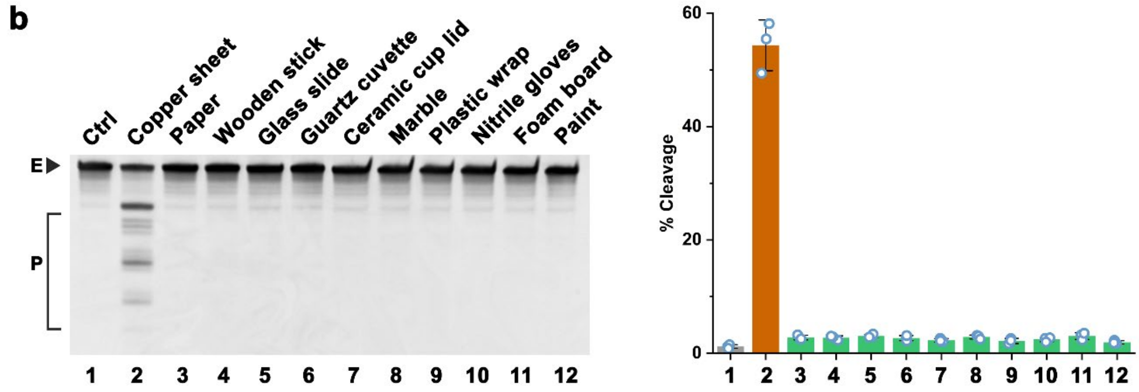

Supplementary Figure 9. The effect of 10 different none-metal surfaces on the PL DNAzyme. a The 10 different none-metal samples. **b** The effect of none-metal surfaces on PL. Composition of the 100 μL reaction system: 0.1 μM PL, none-metal surface and ddH_2O. The reaction temperature and time were set at 23 °C and 15 min, respectively. The E and P represent PL and the cleavage products, respectively. The error bars represented the standard deviations from three repeated measurements.

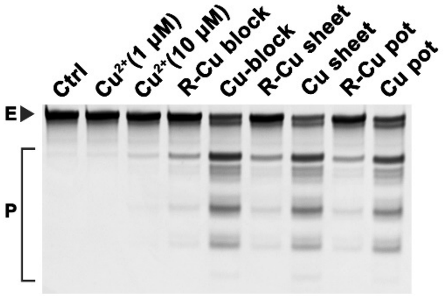

Supplementary Figure 10. The effect of surface-leached (-contacting) solutions on copper surfaces. Composition of the 100 μL reaction system: 0.1 μM PL and ddH_2O on Cu surface (or recovery ddH_2O on Cu surface). The reaction temperature and time were set at 23 °C and 15 min, respectively. The R-Cu block (R-Cu sheet, R-Cu pot) represents copper surface-leached solutions. The Cu block (Cu sheet, Cu pot) represents copper surface-contacting solutions. The E and P represent PL and the cleavage products, respectively.

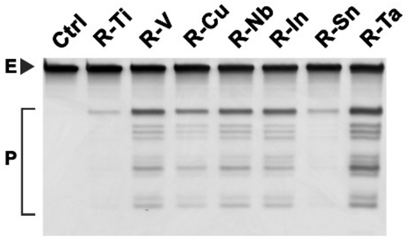

Supplementary Figure 11. Comparative effects of seven metal surface-leached solutions. Composition of the 100 μL reaction system: 0.1 μM PL and recovery ddH_2O on metal surface. The reaction temperature and time were set at 23 °C and 30 min, respectively. The R-Ti (R-V, R-Cu, R-Nb, R-In, R-Sn, R-Ta) represents surface-leached solutions on Ti (V, Cu, Nb, In, Sn, Ta) surface. The E and P represent PL and the cleavage products, respectively.

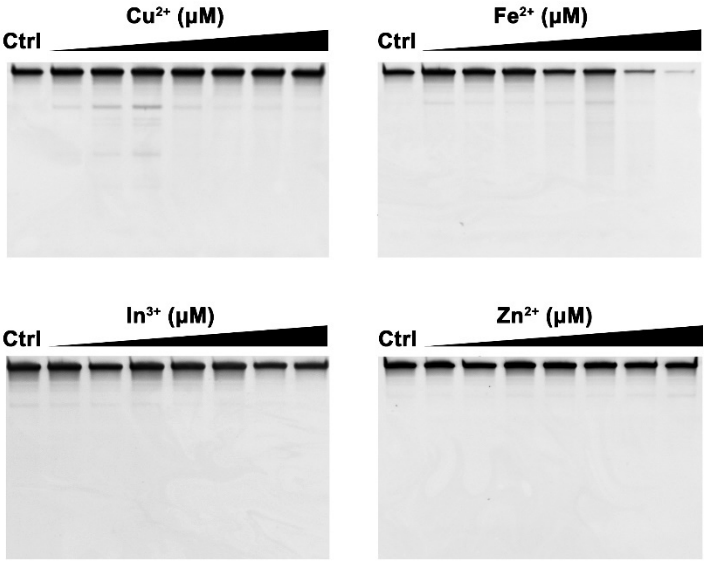

Supplementary Figure 12. The effect of Cu^{2+}, Fe^{2+}, In^{3+} and Zn^{2+} on the PL DNAzyme. Composition of the 100 μL reaction system: 0.1 μM PL, 1 – 1000 μM Cu^{2+} (or Fe^{2+}, In^{3+} and Zn^{2+}) and ddH_2O. The reaction temperature and time were set at 23 °C and 15 min, respectively.

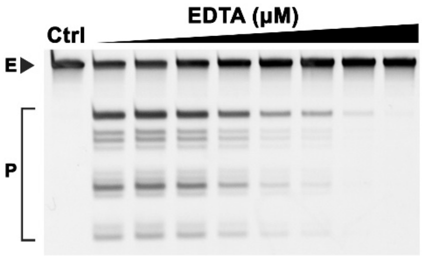

Supplementary Figure 13. The inhibition effect of EDTA on the PL DNAzyme. Composition of the 100 μL reaction system: 0.1 μM PL, 1 – 1000 μM EDTA, Cu surface and ddH_2O. The reaction temperature and time were set at 23 °C and 15 min, respectively. The E and P represent PL and the cleavage products, respectively.

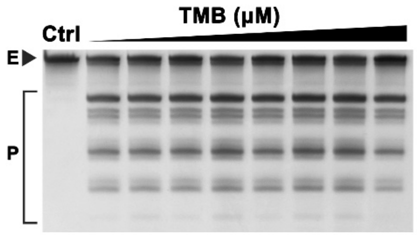

Supplementary Figure 14. The effect of TMB on the PL DNAzyme. Composition of the 100 μL reaction system: 0.1 μM PL, 1 – 1000 μM TMB, Cu surface and ddH_2O. The reaction temperature and time were set at 23 °C and 15 min, respectively. The E and P represent PL and the cleavage products, respectively.

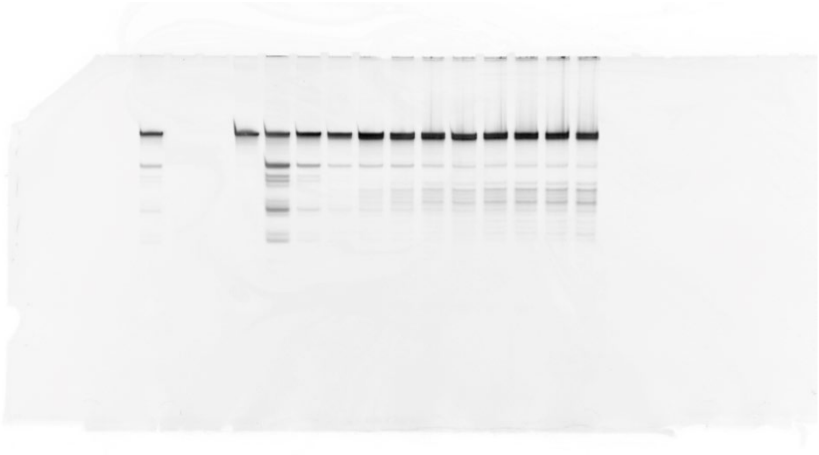

Supplementary Figure 15. The full, uncropped gel image (the left part) of Figure 3d.

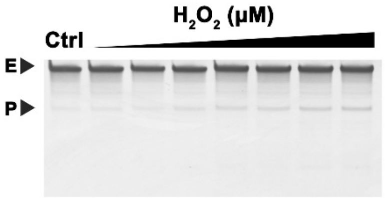

Supplementary Figure 16. The effect of H_2O_2 on the PL DNAzyme. Composition of the 100 μL reaction system: 0.1 μM PL, 1 – 1000 μM H_2O_2 and ddH_2O. The reaction temperature and time were set at 23 °C and 15 min, respectively. The E and P represent PL and the cleavage products, respectively.

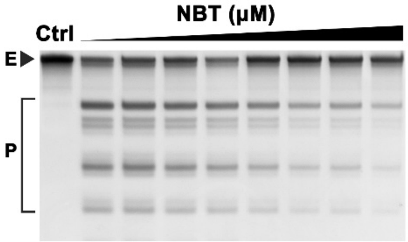

Supplementary Figure 17. The inhibition effect of NBT on the PL DNAzyme. Composition of the 100 μL reaction system: 0.1 μM PL, 1 – 1000 μM NBT, Cu surface and ddH_2O. The reaction temperature and time were set at 23 °C and 15 min, respectively. The E and P represent PL and the cleavage products, respectively.

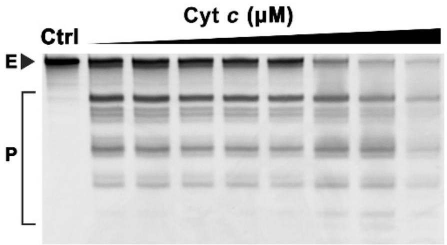

Supplementary Figure 18. The inhibition effect of Cyt *c* on the PL DNAzyme. Composition of the 100 μL reaction system: 0.1 μM PL, 1 – 1000 μM Cyt *c*, Cu surface and ddH_2O. The reaction temperature and time were set at 23 °C and 15 min, respectively. The E and P represent PL and the cleavage products, respectively.

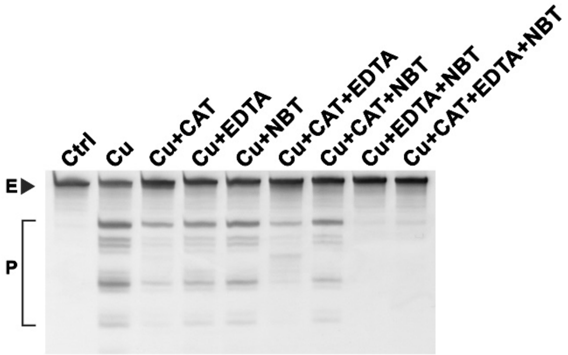

Supplementary Figure 19. The effect of Cu inhibitors on the PL DNAzyme. Composition of the 100 μL reaction system: 0.1 μM PL, inhibitory factors (0.2 U per μL CAT, 100 μM EDTA, 100 μM NBT), Cu surface and ddH_2O. The reaction temperature and time were set at 23 °C and 15 min, respectively. The E and P represent PL and the cleavage products, respectively.

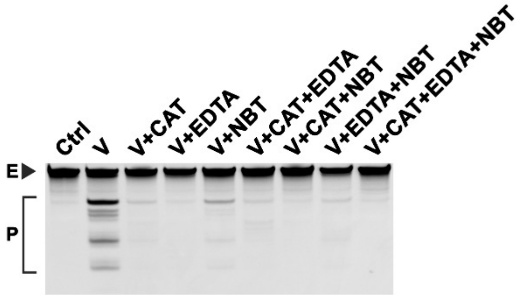

Supplementary Figure 20. The effect of V inhibitors on the PL DNAzyme. Composition of the 100 μL reaction system: 0.1 μM PL, inhibitory factors (0.2 U per μL CAT, 100 μM EDTA, 100 μM NBT), V surface and ddH_2O. The reaction temperature and time were set at 23 °C and 15 min, respectively. The E and P represent PL and the cleavage products, respectively.

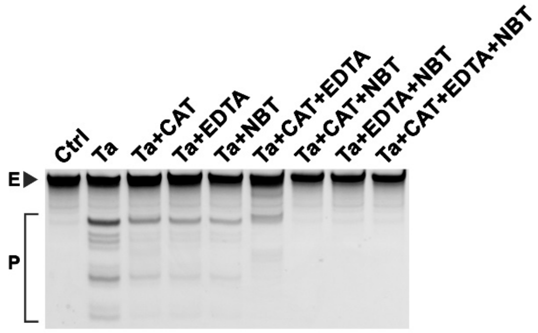

Supplementary Figure 21. The effect of Ta inhibitors on the PL DNAzyme. Composition of the 100 μL reaction system: 0.1 μM PL, inhibitory factors (0.2 U per μL CAT, 100 μM EDTA, 100 μM NBT), Ta surface and ddH_2O. The reaction temperature and time were set at 23 ^oC and 15 min, respectively. The E and P represent PL and the cleavage products, respectively.

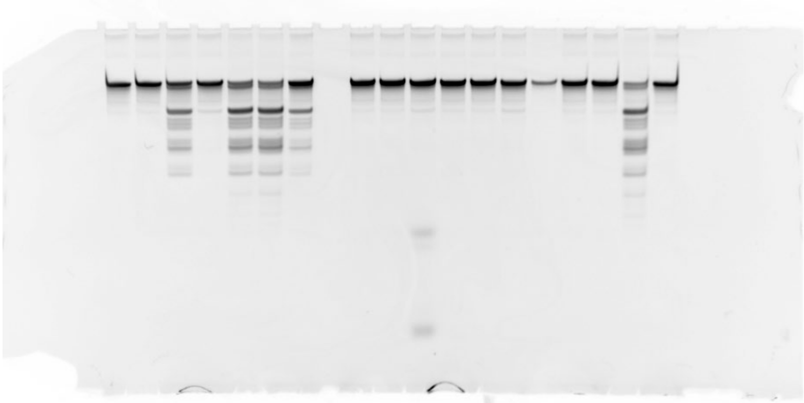

Supplementary Figure 22. The full, uncropped gel image (the left part) of Figure 4a.

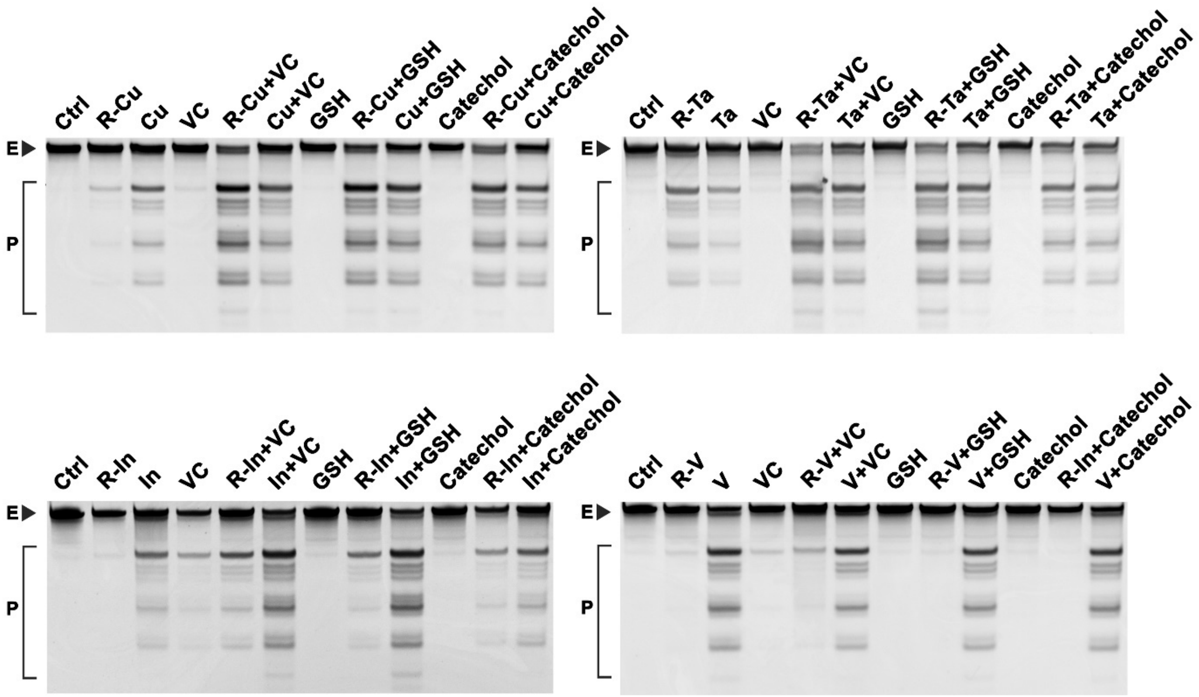

Supplementary Figure 23. The effect of enhancers on the PL DNAzyme. Composition of the 100 μL reaction system: 0.1 μM PL, enhancing factors (100 μM VC, 100 μM GSH, 100 μM Catechol), ddH_2O on metal surfaces (or recovered ddH_2O from metal surfaces after immersion). The reaction temperature and time were set at 23 °C and 15 min, respectively. The R-Cu (R-Ta, R-In, R-V) represents copper (tantalum, indium, vanadium) surface-leached solutions. The Cu (Ta, In, V) represents copper (tantalum, indium, vanadium) surface-contacting solutions. The E and P represent PL and the cleavage products, respectively.

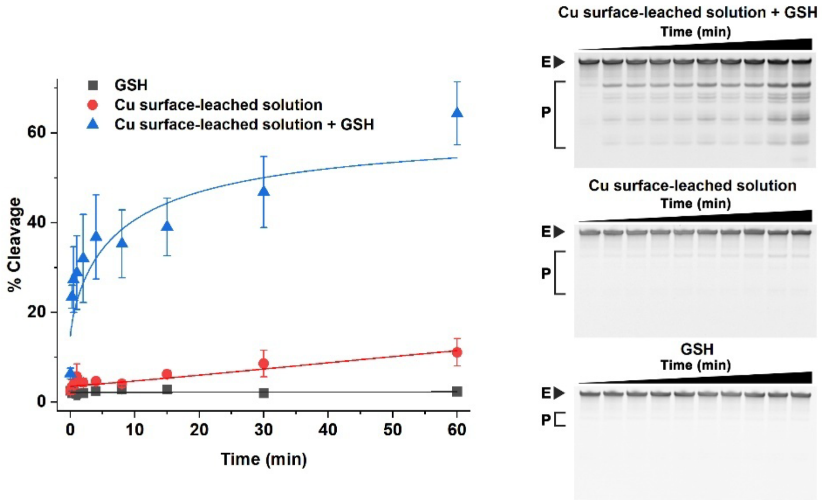

Supplementary Figure 24. Time course of the cleavage reaction of PL in Cu surface-leached solution. The 100 μL reaction system consisted of: 0.1 μM PL, 300 μM GSH, and Cu surface-leached solution (from 0 – 60 min). The reaction was carried out at 23 °C for 15 min. The E and P represent PL and the cleavage products,

respectively. The error bars represented the standard deviations from three repeated measurements.

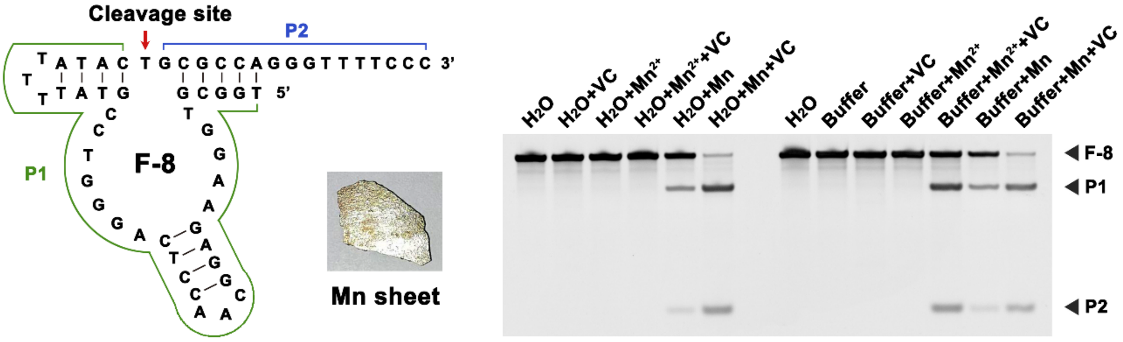

Supplementary Figure 25. The effect of Mn surface on the F-8 DNAzyme. The 100 μL reaction system consisted of: 0.1 μM F-8, 10 mM Mn^{2+}, enhancing factor (300 μM VC), and ddH_2O; 0.1 μM F-8, enhancing factor (300 μM VC), and ddH_2O placed on a Mn surface; 0.1 μM F-8, 10 mM Mn^{2+}, enhancing factor (300 μM VC), and buffer (50 mM HEPES, pH 7.4); or 0.1 μM F-8, enhancing factor (300 μM VC), and buffer (50 mM HEPES, pH 7.4) placed on Mn surface. The reaction was carried out at 23 °C for 60 min. P1 and P2 denote the long and short self-cleavage products of the F-8 DNAzyme, respectively.

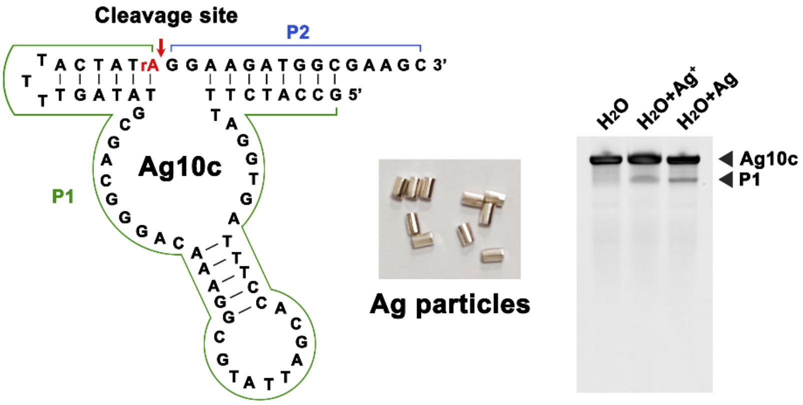

Supplementary Figure 26. The effect of Ag surface on the Ag10c DNAzyme. The 100 μL reaction system consisted of: 0.1 μM Ag10c and ddH_2O placed on an Ag surface; or 0.1 μM Ag10c, 1 μM Ag^+, and ddH_2O. The reaction was carried out at 23 °C for 60 min. P1 and P2 denote the long and short self-cleavage products of the Ag10c DNAzyme, respectively. The P2 fragment was not detected on the 16 % denaturing PAGE gel due to its short length and low yield.

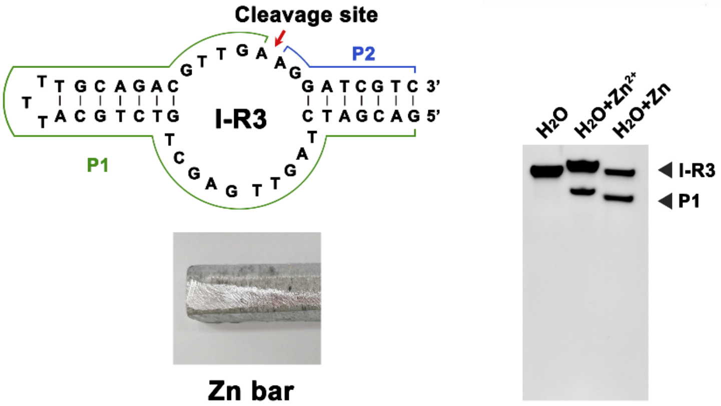

Supplementary Figure 27. The effect of Zn surface on the I-R3 DNAzyme. The 100 μL reaction system consisted of: 0.1 μM I-R3 and ddH_2O placed on an Zn surface; or 0.1 μM I-R3, 50 mM Zn^{2+}, and ddH_2O. The reaction was carried out at 23 °C for 60 min. P1 and P2 denote the long and short self-cleavage products of the I-R3 DNAzyme, respectively. The P2 fragment was not detected on the 16 % denaturing PAGE gel due to its short length and low yield.
