# Supplementary material for: Metal surface-triggered DNAzyme catalysis for efficient DNA cleavage
Source: Commun Chem. 2026 Jan 19;9:91. doi: 10.1038/s42004-026-01893-z (PMC12909895; doi:10.1038/s42004-026-01893-z)
Supplement: Supplementary file 2 — Description of Additional Supplementary Files [file 42004_2026_1893_MOESM2_ESM.docx]

**File:** Supplementary Data 1

**Description:** raw data used to generate Figure 1e, Figure 1f, Figure 2a, Figure 2b, Figure 2c, Figure 3a, Figure 3b, Figure 3c, Figure 3e, Figure 3f, Figure 4b, Supplementary Figure 1, Supplementary Figure 7, Supplementary Figure 9, Supplementary Figure 24.
